# An unusual T-cell childhood acute lymphoblastic leukemia harboring a yet unreported near-tetraploid karyotype

**DOI:** 10.1186/1755-8166-4-20

**Published:** 2011-09-21

**Authors:** Daniela RN Garcia, Samarth Bhatt, Marina Manvelyan, Mariana T de Souza, Renata Binato, Thais F Aguiar, Eliana Abdelhay, Maria Luiza M Silva

**Affiliations:** 1 Cytogenetics Department, Bone Marrow Unit (CEMO), The National Cancer Institute (INCA), Praça da Cruz Vermalha, 23, 6th floor, Centro, 20.230-130, Rio de Janeiro, RJ, Brazil; 2 Jena University Hospital, Institute of Human Genetics, Kollegiengasse 10, D-07743 Jena, Germany; 3 Stem Cells Department, Bone Marrow Unit (CEMO), The National Cancer Institute (INCA), Praça da Cruz Vermalha, 23, 6th floor, Centro, 20.230-130, Rio de Janeiro, RJ, Brazil; 4 Onco-hematology Pediatric Service, The National Cancer Institute (INCA), Praça da Cruz Vermelha, 23, 8 th floor, Centro, 20.230-130, Rio de Janeiro, RJ, Brazil

**Keywords:** T-ALL, childhood, near-tetraploidy

## Abstract

**Background:**

Near-tetraploid (model #81-103) and near-triploid (model #67-81) karyotypes are found in around 1% of childhood acute lymphoblastic leukemia. Due to its rarity, these two cytogenetic subgroups are generally included in the hyperdiploid group (model # > 51). Therefore separate informations about these two subgroups are limited to a few reports. Some studies found that near-tetraploidy is relatively more frequent in higher median ages and it is associated to Frech-American-British Classification subtype L2. Although the mechanisms by which leukemic blast cells divide is still unclear, studies have suggested that hyperdiploidy, near-triploidy and near-tetraploidy do not seem to share the same mechanism.

**Findings:**

Herewith, we present a new childhood T-acute lymphoblastic leukemia case of near-tetraploid karyotype with loss of two p53-gene copies, characterized in detail by cytogenetic and molecular studies.

**Conclusion:**

We suggest that p53 is a good target gene to be screened, once p53 is one of the main effectors of cell cycle checkpoints.

## I. Background

Chromosomal ploidy status has well known prognostic significance implications in childhood acute lymphoblastic leukemia (ALL). In general, model numbers (mn) of more than 50 chromosomes, are a good prognostic factor. Among these, high-hyperdiploidy (mn 51-65) is connected with an excellent outcome. Cases with mn > 65 are rare: near-triploidy (mn 67-81) and near-tetraploidy (NT) (mn 81-103) can be found in only 0.3% and 0.7-2% of childhood ALL, respectively [1,2]. According to literature, near-tetraploidy appears to be associated to French-American-British (FAB) Classification L2 cytomorphology, and patients with karyotypes of this rare cytogenetic subgroups usually present in higher median ages [3].

The immunophenotypic profile comprises both B- and T- cell lines. While, NT in B-cells is correlated to a favorable prognosis, NT in T-cells is discussed controversially in the literature. Pui *et al*. (1990) [3] related NT in T-cell lineage with a poor prognosis, as 9/20 T-ALL cases with NT karyotype, 5 of them relapsed or died despite of the intense chemotherapy. On the other hand, Lemez *et al*. (2010) [2] described a favorable outcome in 36 T-ALL patients.

At cytogenetic level, it is not rare to find structural abnormalities besides the polyploidy, but the frequent fuzzy chromosomal appearance and limitations of standard banding techniques lead to many non-identified chromosomes hampering a detailed cytogenetic analysis [1,2]. In the majority of childhood ALL studies, cases of near-tri- and near-tetraploidy are included in the hyperdiploid group (mn > 51); therefore separate information about these two subgroups are limited to a few reports [1].

It has been described that, p53 gene may lead the cell to apoptosis and cellular senescence [4,5]. Some studies have shown that, in absence of p53, cells with damaged DNA fail to properly respond to this damage checkpoints but instead continue to proliferate, which results in structural abnormalities, aneuploidy and polyploidy including tetraploidy. The p53 gene plays an important role at various checkpoints of the cell cycle, especially in M-phase, where this gene contributes to the control of centrosome duplication, and also to the prevention of DNA duplication when chromosome segregation is impaired by spindle inhibitors [5].

Herewith we present a new T-ALL child case of NT karyotype with loss of two p53-gene copies, characterized in detail by cytogenetic and molecular studies.

## II. Case Presentation

### 1. Case Report

A 15-year-old male was admitted to the Onco-hematology Pediatric Service of The National Cancer Institute (INCA), Rio de Janeiro, Brazil, on January/2011, presenting clinical history of related diffuse bone pain, night sweats, leukocytosis, thrombocytopenia and presence of cells with blastic features.

Physical examination revealed that the patient was flushed, jaundiced, with active bleeding gums, petechiae on the palate. It was observed that the presence of enlarged palpable lymph nodes in the cervical, submandibular, bilaterally axillary and inguinal regions; cardiovascular and respiratory systems were normal. The patient presented hepatomegaly (3 cm below the right costal margin) and splenomegaly (7 cm below the left costal margin). His mother was diagnosed as HIV-positive, as was the patient himself. A diagnostic lumbar puncture was performed after cranial tomography scans and showed no signs of bleeding or masses in the parenchyma.

Laboratory examinations revealed tumor lysis syndrome, thrombocytopenia, hypercellularity, and liquor findings that were consistent with central neural system (CNS) infiltration. Hemoglobin levels were low (11 g/dl), white blood cell (WBC) count was 49 × 10^9^/l with 58% blast cells, platelet count was 41 × 10^9^/l and LDH was 8580 U/l. The morphological evaluation of bone marrow contests showed moderate hypercellularity, presence of lumps and 90% of blast cells with lymphoid characteristics. Flow cytometry revealed a population of blast cells that expressed CD4/8, CD10, CD7, CD2, TDT, TDT/cCD3, which was compatible with T-ALL [6]. Molecular genetic analysis by Reverse Transcriptase Polymerase Chain Reaction (RT-PCR) excluded the presence of ETV6/RUNX1 fusion gene [7].

The patient was treated according to medium risk of BFM 1995 protocol. Peripheral blood of chemotherapy showed good response to treatment at 8^th ^day (D) and bone marrow of D33 showed remission (bone marrow M1). To date, the patient is enrolled on the maintenance phase of chemotherapy.

### 2. Banding Cytogenetics

Standard cytogenetic analysis of the bone marrow was performed. The diagnosis of leukemia was established based on the French-American-British classification (FAB) criteria, including clinical manifestations, immunophenotyping and cytogenetic analysis. Karyotypes were obtained at the time of the case's diagnosis. GTG-banding was done according to standard protocols. Chromosomes were identified and analyzed in accordance with ISCN 2009 [8].

### 3. Molecular Cytogenetics

The NT karyotype was further analyzed by multiplex fluorescence *in situ *hybridization (M-FISH) [9] using all 24 human whole chromosome paints as probes [10]. Additionally, two commercially available probes, centromere specific probe for chromosome 17 (CEP 17) and a locus specific identifier probe for the p53-gene (LSI P53) in 17p13.1 (Abbott/Vysis, Place and name of the probe provider company) were used according to manufacturer's instructions.

## III. Results

**Figure 1:**
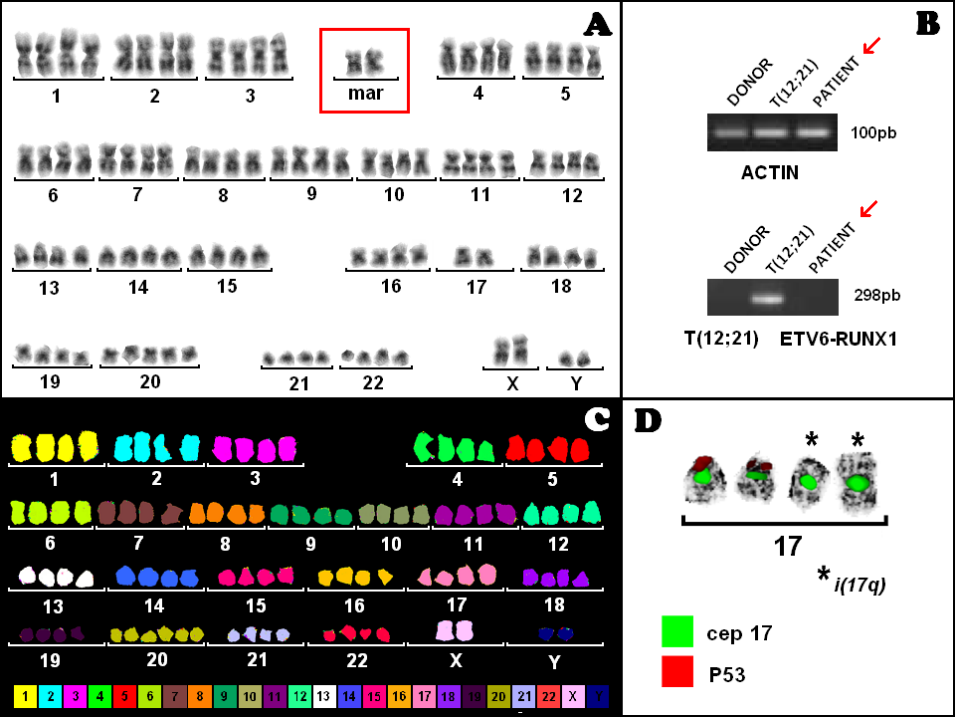
**Cytogenetic and molecular analysis**. **A **Partial and total G-banding karyotype showing a near-tetraploidy (mn 94); **B **RT-PCR results showing ETV6-RUNX1 fusion gene negative. cDNA of a health donor (DONOR) was used as negative (12;21) control for ETV6-RUNX1 fusion **C **M-FISH showing the karyotype: 94, XXYY, i(17)(q10)x2,+20,+20; **D **FISH cep 17 and LSI p53 showing the deletion of 2 copies of P53 gene on derivatives i(17q).

GTG-banding studies at the time of ALL diagnosis revealed a NT karyotype (mn 94), including derivative chromosomes in 15/15 cells (Figure 1A). Since the identification of some chromosomes and molecular abnormalities were not conclusive, molecular cytogenetics M-FISH was applied to further validate the data (Figure 1C). The later, identified derivative chromosomes 17, presenting the loss of two copies of p53 gene, which were further characterized using cep 17 and LSI p53 (Figure 1D).

Overall, the NT karyotype was characterized as 94, XXYY, i(17)(q10)x2,+20,+20.

Furthermore, molecular genetics using RT-PCR excluded the presence of ETV6-RUNX1 fusion gene (Figure 1B).

## IV. Discussion

Although the mechanisms by which leukemic blast cells divide is still unclear some studies have suggested that hyperdiploidy, near-triploidy and near-tetraploidy do not seem to share the same mechanisms. Hyperdiploid leukemic cells generally arise by a simultaneous gain of all additional chromosomes during a single abnormal cell division [7]. Some findings using multicolor banding FISH suggested that near-triploid cases are more likely to arise by random nondisjunction mechanism [11]. Some studies propose that NT is most likely to be a doubling of a near-(pseudo)diploid clone with subsequent chromosome losses rather than a simultaneous acquisition of chromosomes, as seen in high-hyperdiploidy [7].

Abnormal cells, defective checkpoints or cell cycle regulators, as p53 gene, are thought to be responsible to the lack for normal division [5]. Near-tetraploidy observed in our patient is being described in the literature for the first time as it was ETV6/RUNX1 negative and presented a loss of 2 copies of p53gene. These findings corroborate to the mechanisms involved in the formation of this karyotype group that could include genes enrolled in checkpoints. The p53 is one of the main effectors of cell cycle checkpoints, contributing to the qualitative and quantitative maintenance of chromosomal stability of the genetic material. Therefore, we suggest that p53 is a good target gene to be screened for further prospective and retrospective studies in childhood near-tetraploid ALL.

## Consent

This study was approved in the Ethics Committee of The National Cancer Institute (registration number: 077/08). The written informed consent number obtained from the patient's parents for publication of this case report is retained with the Ethics Committee of The National Cancer Institute.

## Declaration of competing interest

The authors report no conflicts of interest. The authors alone are responsible for the content and writing of the paper.

## Authors' contributions

DRNG, MTdS and MLMS conducted the cytogenetic analysis of probes and the karyotype interpretation; SB and MM conducted FISH and further MCB analysis. RB conducted RT-PCR analysis. TFA provided data on the clinical history of the child. All contributors drafted the manuscript. EA and MLMS revised the manuscript critically for important intellectual content and the authors alone are responsible for the content and writing of the paper. All authors read and approved the manuscript
